# Influence of catch up growth on spatial learning and memory in a mouse model of intrauterine growth restriction

**DOI:** 10.1371/journal.pone.0177468

**Published:** 2017-05-24

**Authors:** Cristina Duran Fernandez-Feijoo, Cristina Carrasco Carrasco, Núria Villalmazo Francisco, Judit Cebrià Romero, Jose Ramon Fernández Lorenzo, J. C. Jiménez-Chillaron, Marta Camprubí Camprubí

**Affiliations:** 1 Hospital Álvaro Cunqueiro, Pediatrics Service, Vigo, Spain; 2 Neonatology Service, BCNatal | Barcelona Center for Maternal Fetal and Neonatal Medicine, Hospital Sant Joan de Déu and Hospital Clínic, University of Barcelona, Esplugues de Llobregat, Spain; 3 Institut de Recerca Sant Joan de Déu, Endocrinology, Esplugues, Barcelona, Spain; Bilkent University, TURKEY

## Abstract

**Background:**

Intrauterine growth restriction (IUGR) and rapid postnatal weight gain or catch up growth (CUG) increase the susceptibility to metabolic syndrome during adult life. Longitudinal studies have also revealed a high incidence of learning difficulties in children with IUGR. The aim of the present study was to investigate the effect of nutrition and CUG on learning memory in an IUGR animal model. We hypothesized that synaptic protein expression and transcription, an essential mechanism for memory consolidation, might be affected by intrauterine undernutrition.

**Methods:**

IUGR was induced by 50% maternal caloric undernutrition throughout late gestation. During the suckling period, dams were either fed *ad libitum* or food restricted. The pups were divided into: Normal prenatal diet-Normal postnatal diet (NN), Restricted prenatal diet- Normal postnatal diet + catch up growth (RN+), Normal prenatal diet-Restricted postnatal diet (NR) and Restricted prenatal diet-Restricted postnatal diet (RR). At 4 weeks of age, memory was assessed via a water maze test. To evaluate synaptic function, 2 specific synaptic proteins (postsynaptic density-95 [PSD95], synaptophysin) as well as insulin receptors (IR) were tested by Western Blot and quantitative polymerase chain reaction (qPCR). Brain-derived neurotrophic factor and serum insulin levels were also studied.

**Results and conclusions:**

The RN+ group presented a learning curve similar to the NN animals. The RR animals without CUG showed learning disabilities. PSD95 was lower in the RR group than in the NN and RN+ mice. In contrast, synaptophysin was similar in all groups. IR showed an inverse expression pattern to that of the PSD95. In conclusion, perinatal nutrition plays an important role in learning. CUG after a period of prenatal malnutrition seems to improve learning skills. The functional alterations observed might be related to lower PSD95 activity and a possible dysfunction in the hormone regulation of synaptic plasticity.

## Introduction

Intrauterine growth restriction (IUGR) is the failure of the fetus to reach its growth potential, due to placental, maternal or fetal factors [1]. IUGR affects 3–7% of all newborns [2] and is associated with the development of metabolic diseases [3,4] and neurocognitive problems [5,6,7] during adulthood. There is a growing body of evidence that nutritional or vascular alterations during critical windows of development may lead to IUGR, with lasting effects on cellular structure and function, increasing the risk of developing chronic diseases [8].

The fact that nutrition during early life may have an impact on the development of later diseases, led to the concept of metabolic programming or nutritional imprinting. Barker et al. observed that birthweight (BW) was inversely correlated with cardiovascular disease risk in adulthood. Environmental factors, particularly nutrition, during the fetal period may produce a long-lasting impact on adult phenotypes, programming later diseases such as hypertension, impaired glucose tolerance, type 2 diabetes and obesity [9,10]. On the other hand, accelerated postnatal weight gain during the first 1–2 years of life, known as catch up growth (CUG), is common in IUGR infants. CUG growth is defined as “weight velocity above the normal statistical limits for age and/or maturity during a defined period of time following a transient period of growth inhibition” [11]. The index used for CUG during the first year of life is weight because length is a very variable measurement. It culminates with the individual reaching its original, pre-growth-restriction growth curve.

Human studies have reported that CUG in IUGR children predisposes these children to the development of metabolic diseases in adulthood [11, 12, 13]. Many human and animal studies have supported these findings [14, 15, 16].

The idea that CUG or growth acceleration has adverse effects on long-term health has generated much debate. Faster postnatal growth may have short-term benefits but increases the long-term risk of diabetes, obesity and metabolic disease.

The association between IUGR and long-term neurodevelopmental dysfunction has been extensively described [17,18]. Likewise, IUGR has been related to a reduction in intelligence quotients, difficulties in creative problems solving, attention and executive functions, visuomotor organization and higher order verbal skills [19]. These neurobehavioral and cognitive impairments seem to persist until late childhood and adolescence. Some authors even suggest that these impairments could have a role in many psychiatric disorders in adulthood [20]. On the other hand, recent epidemiological studies suggest that a positive CUG could have a beneficial effect on neurodevelopment in that IUGR babies that exhibited positive CUG had better psychomotor and mental development than those that remained at <10^th^ percentile [21,22]. In the same line, there is growing evidence that positive CUG improves academic achievements in these children [19]. Consequently, the optimal pattern of postnatal growth (to achieve both appropriate metabolic and cognitive health) is unclear and is likely to differ in different populations [12].

The hippocampus is a brain structure that plays an important role in memory and learning. Hippocampal neurons are very sensitive to insults such as ischemia and undernutrition, two situations closely related to IUGR. Perinatal undernutrition affects hippocampal synaptic plasticity [23,24,25] decreasing cell numbers in the main areas (CA1 and CA3) [26,27]. To our knowledge, there is no molecular evidence relating CUG to hippocampal structure and, consequently, learning and memory process.

The aim of this present study was to evaluate the effects of nutrition and positive CUG on memory and learning using an IUGR mouse model. Accordingly, we analysed some of the most important proteins and neuroendocrine receptors involved in the synaptic process.

## Materials and methods

### Animal care and experimental protocol

The experimental procedures were approved by the local ethical committee of the University of Barcelona, following European (2010/63/UE) and Spanish (RD 53/2013) regulations for the care and use of laboratory animals.

**Fig 1:**
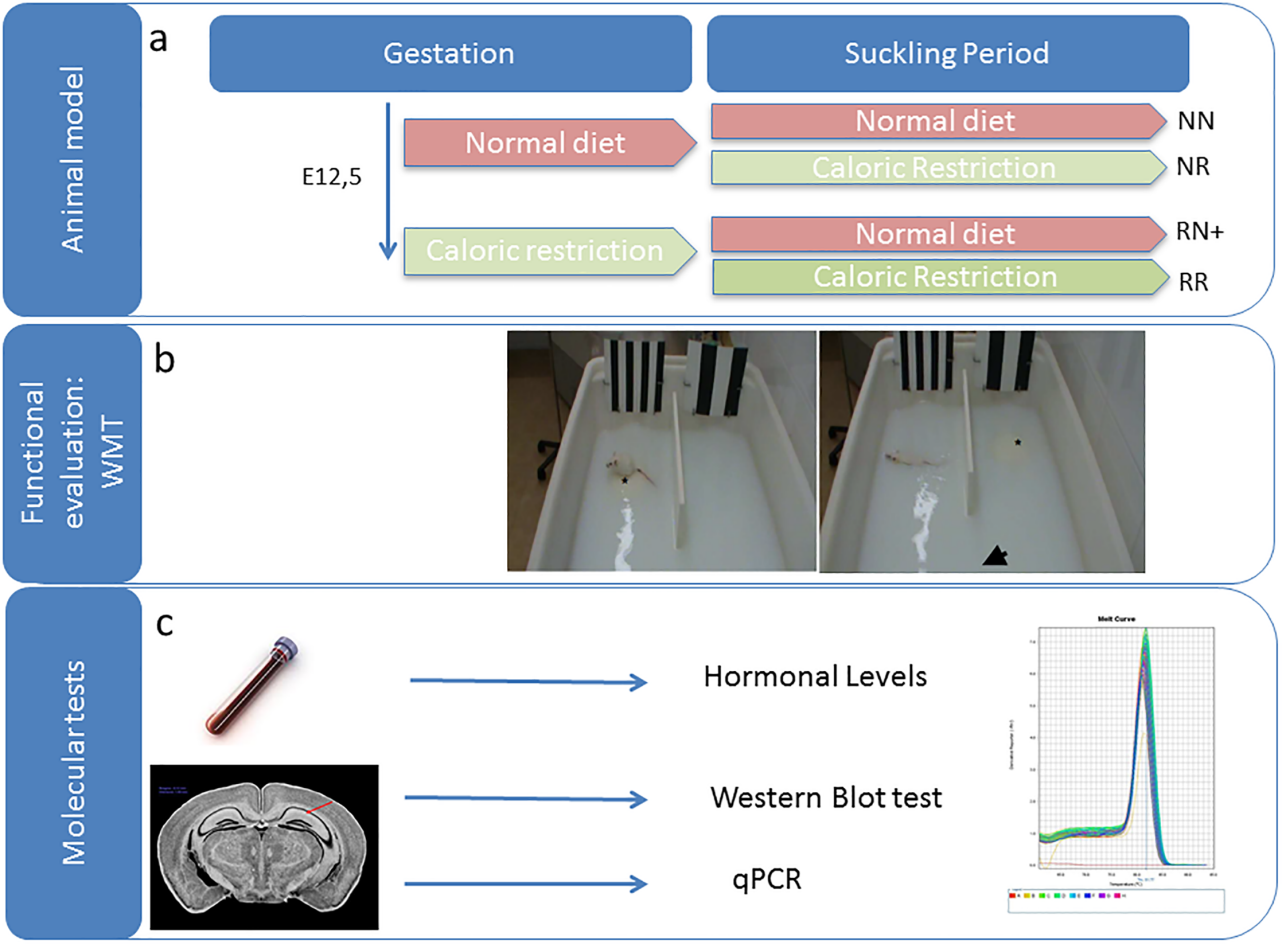
Graphical scheme of the experimental groups and procedures. A) **Animal model:** The diagram of the experiment is represented, with the different groups. B) **Functional evaluation:** The WMT is performed from 25 to 35 days. **C) Molecular tests:** Different test are performed for the molecular evaluation: Hormonal tests (ELISA), Protein quantification (Western blot) and RNA quantification (qPCR).

The Institutional Animal Care and Use Committee approved the animal protocols used in the present study. Six- to eight-week-old female ICR mice were caged with ICR males. Pregnancy was dated with vaginal plugs (day 0.5). Females were subsequently housed individually with free access to food. On pregnancy day 12.5, the females were randomly assigned to a normal diet group (N) or a group subjected to restricted diet (R) (Fig 1A). There were no differences in weight between N and R mothers before pregnancy or at the time of group assignment. From day 12.5 to 18.5 the food intake of R mothers was restricted to 50% of that of the normal diet. The animal food was weighed daily to ensure the appropriate doses for each group. After delivery, the litter size was equalized to 8 in each group. The N and R litters were further distributed into two additional experimental groups; the mothers in both the normal diet and restricted diet groups were provided with either: (1) free access to chow after delivery, or (2) 50% food restriction per day, from the day of delivery until the day of weaning (21 days postpartum). Therefore, there were 4 experimental groups: the NN or control group of mice maintained on a normal diet both pre-natally and post-natally (N = 17), the NR group of mice with normal prenatal diet but postnatal diet restriction (N = 12), the RN+ group of mice with prenatal diet restriction and normal postnatal diet showing CUG (N = 14), and the RR group of mice with both pre- and postnatal diet restriction (N = 13). In all the groups, the pups were weaned at 3 weeks of age. Female litters were excluded to avoid hormonal influences. Males were housed in groups of 5 individuals per cage, separated according to treatment with free access to food and water (S1 Fig).

Body weight was recorded weekly from birth to 1 month of age. Specific growth curves were calculated. IUGR was defined as a BW significantly below that of the NN group, and CUG was considered as weight gain up to the average weight of the NN group during the first month of life.

### Water maze test

Evaluation of spatial memory was carried out with a version of the Morris water maze (MWM) test. The MWM test is widely used in behavioural neuroscience to study spatial learning and memory [28]. It is a test of hippocampus-dependent spatial memory [29]. The rodents were placed in an open pool and the latency to escape (EL) was measured by up to six trials a day for 2–20 days. The pool used was for small animals, being 63 cm in length x 43 cm in width x 35 cm in height, with the water temperature set at 22–23°C, and the water was made opaque by latex suspension. An 8x8-cm plexiglass platform, onto which the rat could escape, was positioned in front of one of the points marked 1 cm below the water surface (Fig 1B). The EL was defined as the time taken to reach the platform and was measured during each trial as an indicator of learning. At two weeks of age, all the pups from each experimental group had been trained four times a day for 19 consecutive days. Each animal had a stimulus assigned for the entire test.

On day 1, each rat was placed in the pool for 50 seconds to become habituated to the training environment. From the second day to the end, the marker in which the platform was located remained constant for each animal, varying depending on the animal and the marker assigned.

The latency from immersion into the pool to escape onto the platform was recorded for each trial, and the observer also manually recorded the route taken by the rat to reach the platform.

On mounting the platform, the rats were given a 10-second rest period, after which the next trial was started. If the rat did not find the platform in 50 seconds, it was manually placed on the platform for a 10-second rest.

The day after finishing the test the animals were sacrificed.

### Blood samples

At 45 days of life, serum insulin values were measured in 5 μl serum samples using an ELISA test (Crystal Chem, Downers Grove, IL, USA) (Fig 1C).

### Western blot analysis

After the MWM test, the pups were sacrificed by decapitation and their brains were rapidly removed, weighed and frozen. Protein extracts from the hippocampus were separated with SDS-PAGE and electro-transferred to a nitrocellulose membrane. The membranes were blocked with 5% nonfat dry milk in tris-buffered saline and incubated first with primary antibodies that recognize PSD95 (1:2000, Abcam), synaptophysin (1:1000, Dako) and insulin receptor (1:500 Santa Cruz Biotechnology, San Diego, CA, USA) overnight at 4°C, and then with their corresponding secondary HRP-conjugated antibodies. Protein signal was detected using the ECL chemiluminescent system (Amersham, Buckinghamshire, UK) (Fig 1C).

### Quantitative real-time PCR

Complementary DNA (cDNA) was generated from 1-μg RNA using oligo-dT primers (Takara Bio, Mountain View, CA, USA) and used for PCR with SYBRGreen (Applied Biosystems, Foster City, CA, USA). Expression was normalized to cyclophilin or TATA box binding protein, as indicated. Primer sequences are available upon request.

### Statistical analysis

Eight animals per group were needed to compare the effects of normal nutrition or undernutrition differences on the MWM test results (assumed risk of 5% and a power of 85%).

Characteristics of the animals in the experimental groups are presented as mean and standard error of the mean (mean ± SEM), and differences between groups were analysed using one-way ANOVA followed by the Tukey post-hoc test in parametric variables. Plasma insulin levels were analysed using a non-parametric test (krusskall-wallis).

The EL of the mice in the MWM test was analysed using two-way analysis of variance (ANOVA) with repeated measures, the factors being treatment and training day, and corrected with Bonferroni assessment. A p value <0.05 was considered significant. All analyses were performed using SPSS 17.0 for Windows.

The methods are summarized in Fig 1.

## Results

### Weight evolution and plasmatic insulin levels

**Fig 2:**
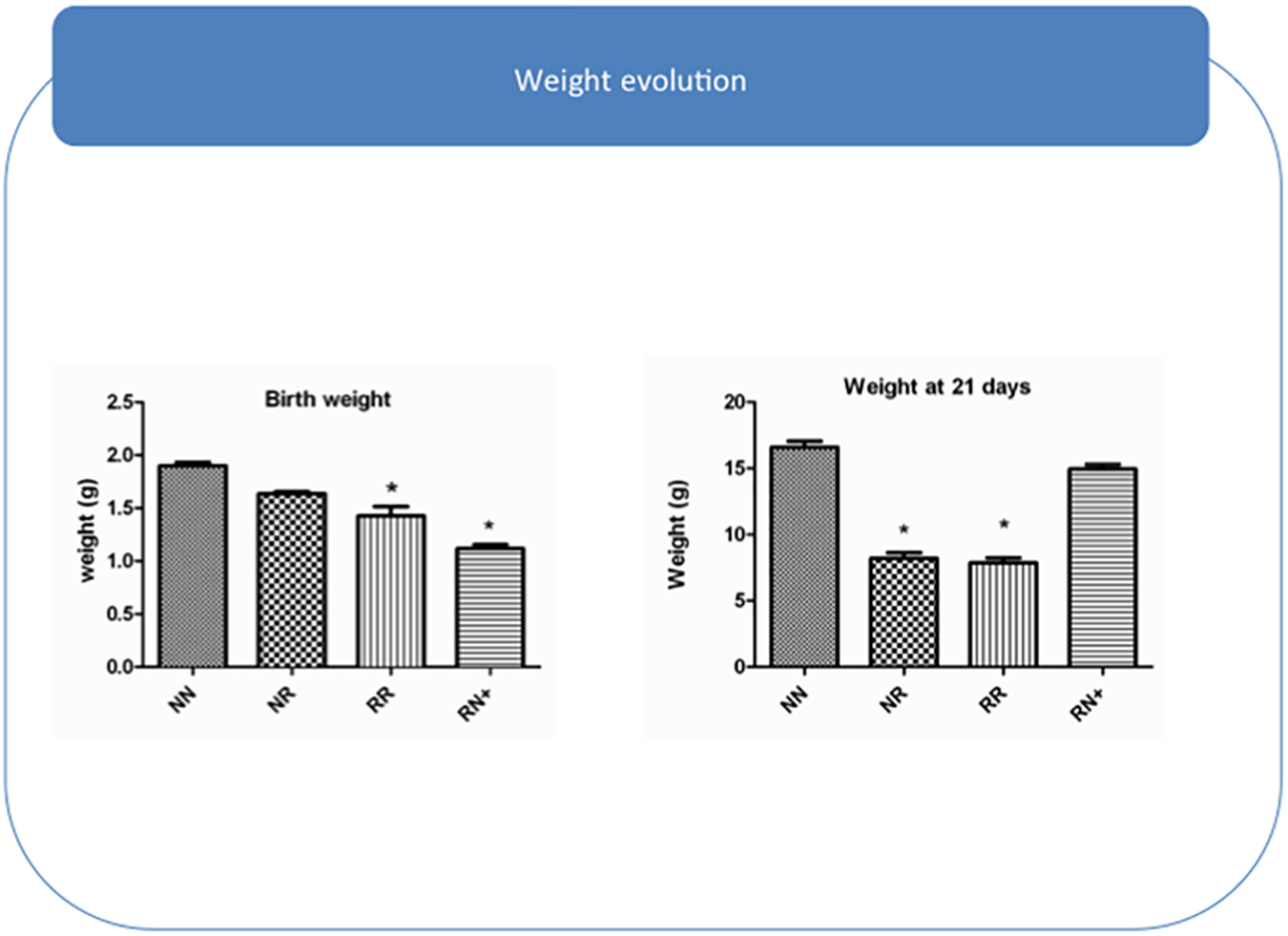
Pup growth evaluation. Pup weight two days after birth, by group. Pup weight at 21 days of life, by group.

The animals were divided into 4 groups: NN (n = 17), NR (n = 12), RN+ (n = 14) and RR (n = 13). Caloric restriction during the last week of gestation significantly reduced offspring BW (RN+ and RR *vs*. NN mice, p<0.001) (Fig 2A). At 3 weeks of life, RN+ mice exhibited CUG and reached a similar body weight to that of NN mice. In contrast, postnatal weight gain was significantly reduced in pups with postnatal food restriction (p<0.01 NN *vs*. RR and NR; p<0.01 RN+ *v*. NR and RR) (Fig 2).

The RR animals showed a lower growth velocity than the NN control mice. The growth velocity of the NR group increased once they had free access to food (at 21 days of life). Nevertheless, they did not reach the average weight of the NN mice (Fig 2). These results are consistent with previously reported data [16].

**Fig 3:**
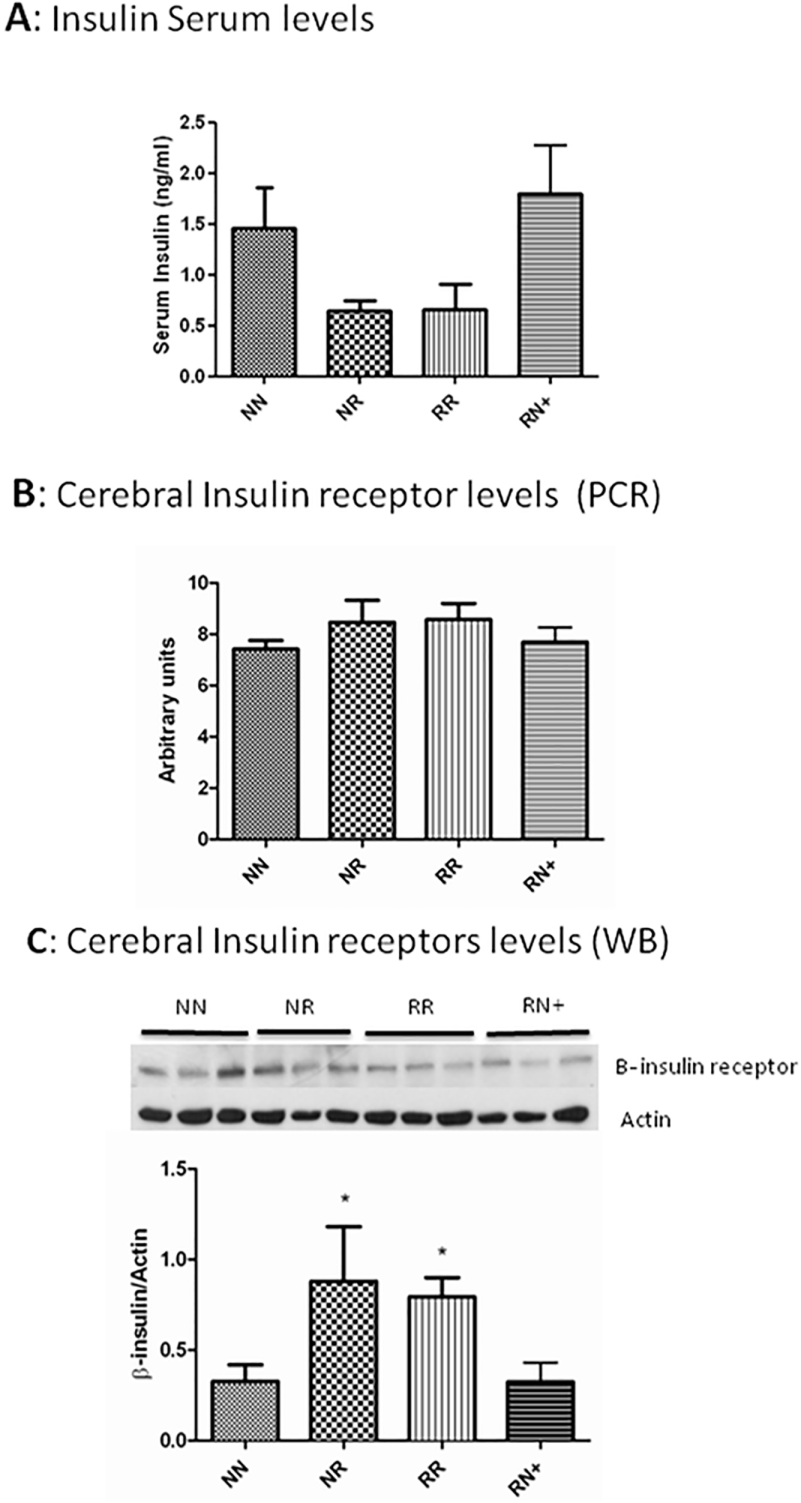
Insulin metabolism. **A**. Serum insulin levels were increased in NN and RN+ animals. Significant differences between groups *p < .05. **B.** Brain insulin receptor transcription was increased in RR and RN animals. **C.** Brain insulin receptor expression was increased in RR and NR animals. Significant differences compared to NN and NR+ *p < .05.

In order to evaluate endocrinological homeostasis, plasma insulin levels were evaluated at the time of sacrifice (45 days). Plasma insulin levels were different among the four groups (p = 003). Despite that, when post-hoc analysis was performed, no statistical significant differences were found (Fig 3A).

### Learning test results

**Fig 4:**
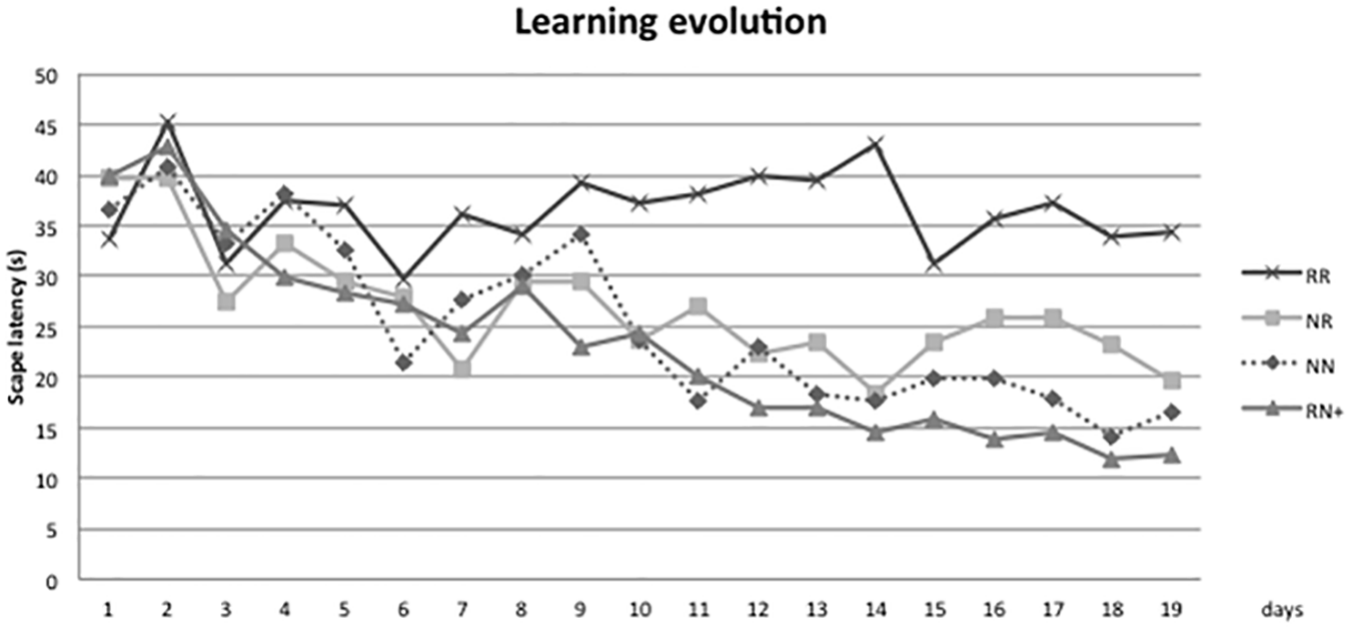
Learning progression. Plot representing average escape latency performed each day by the different groups. There is a difference in the learning process (p<0.01). RN+ animals presented a learning curve similar to that of the NN animals. RR animals showed the worst performance in the test (p <0.05).

Learning and memory were evaluated using the MWM test (Fig 4). The daily EL was averaged from four training trials per day during 19 days and analysed via two-way ANOVA (time and group). Initially, all the groups showed a shorter EL throughout the test, demonstrating that there was a learning process (p<0.01). In the post-hoc analysis, differences were found between NN-RR (p = 0.036) and RN+ -RR (p = 0.016). When the NR group was compared to the RR group, no statistically significant differences were found (0.142). Neither were significant differences found on comparing the NR group with the NN or RN+ groups (P = 1, P = 1).

However, the RR group that did not present CUG showed the worst performance in learning tasks. These data suggest that the memory and learning skills of individuals with prenatal malnutrition could improve with accelerated postnatal growth.

### Synaptic evaluation (brain-derived neurotrophic factor and synaptic proteins) and hormonal receptor level expression

**Fig 5:**
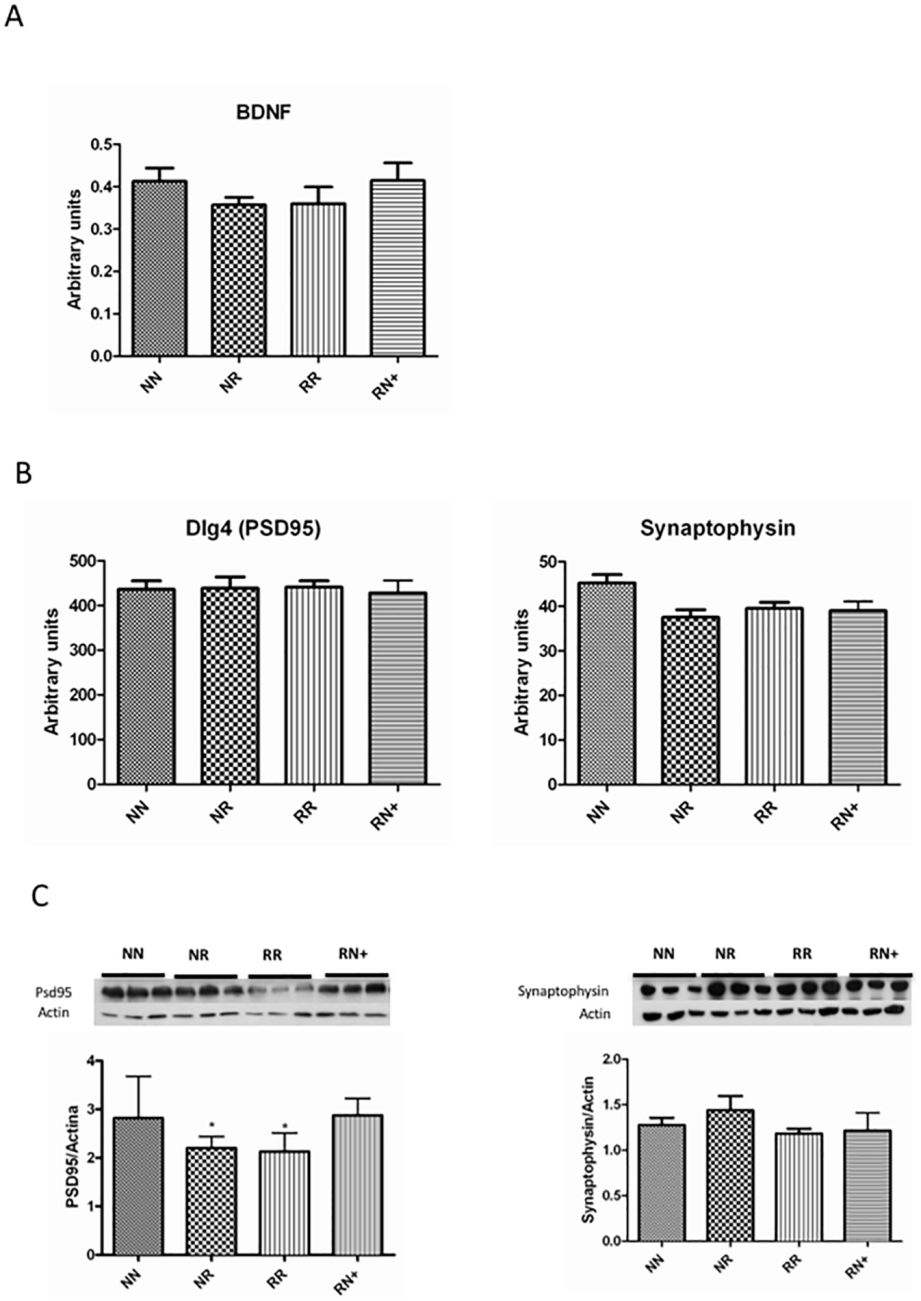
BDNF and synaptic protein expression. A. BDNF mRNA expression was increased in the groups with better learning results. B. PSD95 and synaptophysin transcription. C. PSD95 and synaptophysin expression. There was a reduction in RR PSD95 expression. Significant differences compared to NN and NR+ *p < .05. No differences were observed in synaptophysin expression or trasncription.

Brain-derived neurotrophic factor (*Bdnf*) has emerged as one of the most important molecules involved in memory. In the present study, *Bdnf* expression was reduced in RR (10%) and NR (15%) mice compared to NN and RN+ mice. Thus, Bdnf expression seems to have a tendency to increase in the groups with better learning results (Fig 5A).

Some of the proteins most often studied to evaluate synapsis function are postsynaptic density-95 (PSD95) and synaptophysin. Analysis of synaptic protein levels in the hippocampus indicated a significant reduction in PSD95 (or Dlg4) in RR mice compared to NN and RN+ animals (p<0.05). However, there were no significant differences in synaptophysin levels (Fig 5B). Interestingly, there were no significant changes in mRNA expression of *PS95* (*Dlg4*) and synaptophysin, suggesting post-transcriptional regulation.

The brain insulin receptor has been related to memory and learning. Insulin is considered a potential cognitive enhancer as it modulates synaptic plasticity and dendritic morphology, which are two processes necessary for learning and memory formation. RR individuals presented significantly higher levels of insulin receptor expression compared to the other groups (Fig 3B and 3C), there seem to be the same tendency between transcription and protein expression.

## Discussion

According to the early origins hypothesis of Baker [9], IUGR increases the risk of the development of chronic disease later in life. Fetal programming implies that adverse environmental conditions during critical periods of prenatal growth may result in long-lasting changes in metabolism and other essential structures. These changes allow fetal survival, but they also have an impact on the future life of the fetus. Specifically, fetal growth restriction has been linked to the development of metabolic syndrome and neurocognitive disabilities, including learning alterations and temporal-spatial memory deficits [30,31,32]. The pathophysiology is not well defined, but it has been suggested that growth restriction could induce changes in the regulation of gene expression, leading to the development of adverse outcomes later in life [10].

A significant number of individuals with IUGR present CUG. Animal and epidemiological studies suggest that accelerated postnatal growth contributes to the development of metabolic syndrome [34,35,]. In a previous study comparing IUGR mice with and without CUG the former mice developed glucose intolerance and obesity while the latter mice did not [16]. Thus, from an endocrinological point of view it seems reasonable to propose that early CUG should be avoided in order to prevent late onset metabolic dysfunction. However, the role of CUG in neurodevelopmental outcome still remains unclear.

Catch up growth is most commonly seen after birth in infants with a low BW, being a global problem affecting over 20 million newborns a year. As it has been demonstrated, faster postnatal growth may have short-term benefits but increases the long-term risk of aging, obesity and metabolic disease.

On the other hand, some authors have associated CUG in IUGR with a better neurocognitive developmental outcome [33]. Consequently, the optimal pattern of postnatal growth is unclear and most likely differs in different populations [34,35].

Therefore, early counseling for controlling early rapid growth during the first months of life, when feeding patterns are strongly influenced by the infant and growth is regulated by nutrition, could, to some extent, counterbalance the ominous growth trajectory. Long-term interventional studies in pregnancies at high risk of low-BW delivery as well as on postnatal growth of low-BW newborns are needed to determine which strategy would be the most feasible in practice [36].

Current nutritional strategies that promote CUG should include the monitoring of weight-for-length and adiposity, and future research should seek to define “healthy CUG”[37,38].

We based our study upon a previously described mouse model of IUGR and CUG [39]. First, we confirmed that IUGR mice presenting postnatal CUG developed some features of the metabolic syndrome: increased adiposity and hyperinsulinemia [16,37]. These data suggest that abnormalities in insulin secretion and/or clearance can be modulated by a postnatal dietary intervention [16]. However, our results show that memory and learning skills can improve with accelerated postnatal growth. These findings are also in line with human epidemiological studies, which suggest that IUGR children with CUG show better learning skills, although they present an increased risk of metabolic problems [34]. In the same line, there are other studies that point to the importance of CUG in behavioural difficulties [40,41].

Another major point of discussion is the evolution of the animals with normal prenatal nutrition but subjected to undernutrition in the suckling period. This NR group had better outcomes than the RR group, but remained worse than the animals with a normal postnatal nutrition. These findings emphasize the importance of nutrition during infancy, which is also a critical period in brain development. Indeed, there are numerous animal and human studies that have demonstrated this [42, 43, 44].

However, the prenatal undernutrition group that did not present CUG (the RR group) showed the worst performance in learning tasks, showing the effects of both pre- and postnatal undernutrition. Despite that, it is important to note that the animals with a restricted prenatal diet followed by normal nutrition after birth (RN+) tended to have similar learning curves to those of normal animals. All these data support the increasing evidence that nutrition during the postnatal period is essential for neurodevelopmental outcome [42,43,44]. This hypothesis is also supported by the results of the NR group. Animals with normal prenatal nutrition randomized to receive postnatal undernutrition, presented some learning disabilities, although perhaps not as relevant as those of the RR animals. Nonetheless, their learning and memory process was different to that of the control NN group and that of the animals receiving normal postnatal nutrition.

Although the neuropathological bases of learning and memory disabilities in IUGR babies are not clear, the hippocampus, which is an essential structure in learning and memory processes, appears to be particularly susceptible to undernutrition and chronic placental insufficiency, both of which are related to fetal growth. Some animal studies have shown that these adverse perinatal conditions particularly affect CA1 pyramidal neurons, decreasing excitatory synaptic input and reducing dendritic spine density [43, 44].

Neurotrophins are an important group of molecules involved in the regulation of synapses and brain growth. They are crucial mediators in the facilitation of brain connectivity, neuronal plasticity, synaptic integrity and promotion of basal neurogenesis [45], and BDNF is one of the neurotrophins most frequently studied. It plays an essential role in regulating hippocampal neurogenesis, synaptogenesis and memory consolidation.

The role of BDNF in the early phase of long term potentiation (LTP) is likely dependent on the post- translational regulation of pre-post-synaptic proteins [46]. In our study, the animals with the best results in the MWM test presented increased levels of BDNF. These results are consistent with other behavioural studies suggesting that altered BDNF signalling in the hippocampus and cortex has a significant impact on cognitive functions [47, 48, 49, 50].

In excitatory synapses, the PSD95 protein is anchored in the post-synaptic compartment and acts as a scaffolding protein, being necessary for AMPA and NMDA glutamate receptor traffics and transynaptic signalling. It is considered key in modulating excitatory synaptic strength and plasticity [51,52]. The NN and RN+ mice presented similar learning curves and similar levels of PS95, both of which were higher when compared to NR and RR mice. These results are consistent with recent findings that suggest that PSD95 may have a role in synaptic maturation and functionality [53], as well as being a crucial factor driving AMPA receptor incorporation during LTP and experience-driven synaptic strengthening [54]. Moreover, reduced PSD95 expression, as in RR mice, has been described in animals exposed to perinatal hypoxia, and has also been associated with impaired spatial learning and memory [55]. In our mouse model, there is an equivalent pattern of expression in BDNF and PSD95, suggesting a possible regulation of PSD95 expression through BDNF [56]. Nonetheless, it should be taken into account that the changes in PSD95 were only in the expression but not in the transcription (no differences in levels of Dig4 were detected). In support of this hypothesis, several studies have shown that BDNF modulates the expression of many pre- and postsynaptic proteins [56,57,58].

Regarding synaptic function at the presynaptic level, synaptophysin plays a role in synaptic vesicle biogenesis and trafficking and has been used as an indicator of the total pool of synaptic vesicles [59]. In our study, there were no differences in synaptophysin expression among groups. These results indicate that the total number of synapses and/or synaptic vesicles might not be altered. Therefore, the differences between RR mice and NN/ RN+ mice might be due to functional defects rather than to the total number of synapses.

The regulation of the formation of synapses and their functionality is a complex process. Several studies have argued that hormones like insulin may play an important role in modulating learning and memory formation. Indeed, several studies have demonstrated beneficial effects of insulin, improving hippocampus-dependent memory [60, 61, 62]. Based on this, we analysed insulin receptor (IR) expression in the hippocampus after spatial learning. Interestingly, we found that the RR group showed the highest levels of the IR protein compared to the animals that exhibited successful learning (NN, RN+, NR). These findings are consistent with data reported by Dou et al. in rats with diabetes mellitus. Following the MWM test, the gene expression of IR showed an up-regulation in the CA1, but a down-regulation in the CA3 region in animals with learning problems. This was associated with a significant reduction in hippocampal IR protein levels [63].

Regarding the relation between insulin and PSD95, Lee CC et al. demonstrated that the stimulation of rat hippocampal slices with insulin increases PSD-95 protein expression in the hippocampal CA1 area in a PI3K-dependent manner, providing evidence that insulin could also modulate hippocampal synaptic plasticity through the regulation of PSD95 [64]. In our study, the groups with successful learning showed an inverse correlation in protein expression of IR and PSD95. Moreover, the animals with better learning results presented higher levels of plasma insulin, lending support to the idea of an improvement in learning with insulin [62, 63].

The results of, our study provide combined biochemical and neurobehavioral data to show that CUG improves learning skills in a murine mode of IUGR. At the molecular level, this could be related to an increase in BDNF expression, which has been reported to have an important role in synaptic protein regulation, including PSD95. Likewise, hormonal modulation of synaptogenesis is also important. Insulin has been described to be involved in memory processes in neurodegenerative diseases [65, 66, 67]. In this regard, our results provide new evidence that hormonal modulation during the earliest periods of brain development is also essential. Hence, animals with normal or increased plasma insulin levels showed better results in the memory test.

In summary, our results strengthen the hypothesis that postnatal CUG following IUGR is critical for memory consolidation in animal models. However, it is well known that CUG is also related to increased risk of metabolic dysfunction. Taking all of this into account, we consider that the real challenge is to find the correct balance of early weight gain in the IUGR population so as to prevent obesity and diabetes on one hand and learning disabilities on the other.

## Supporting information

S1 Fig Food intake was monitored daily in NN females during gestation and lactation. R+ and RR females received 50% of the food consumed compared to the average quantity received by the NN females.
(TIF)

S2 Fig Data for experiments. (XLS)
