# Supplementary figures and images for: Influence of catch up growth on spatial learning and memory in a mouse model of intrauterine growth restriction
Source: PLoS One. 2017 May 24;12(5):e0177468. doi: 10.1371/journal.pone.0177468 (PMC5443512; doi:10.1371/journal.pone.0177468)

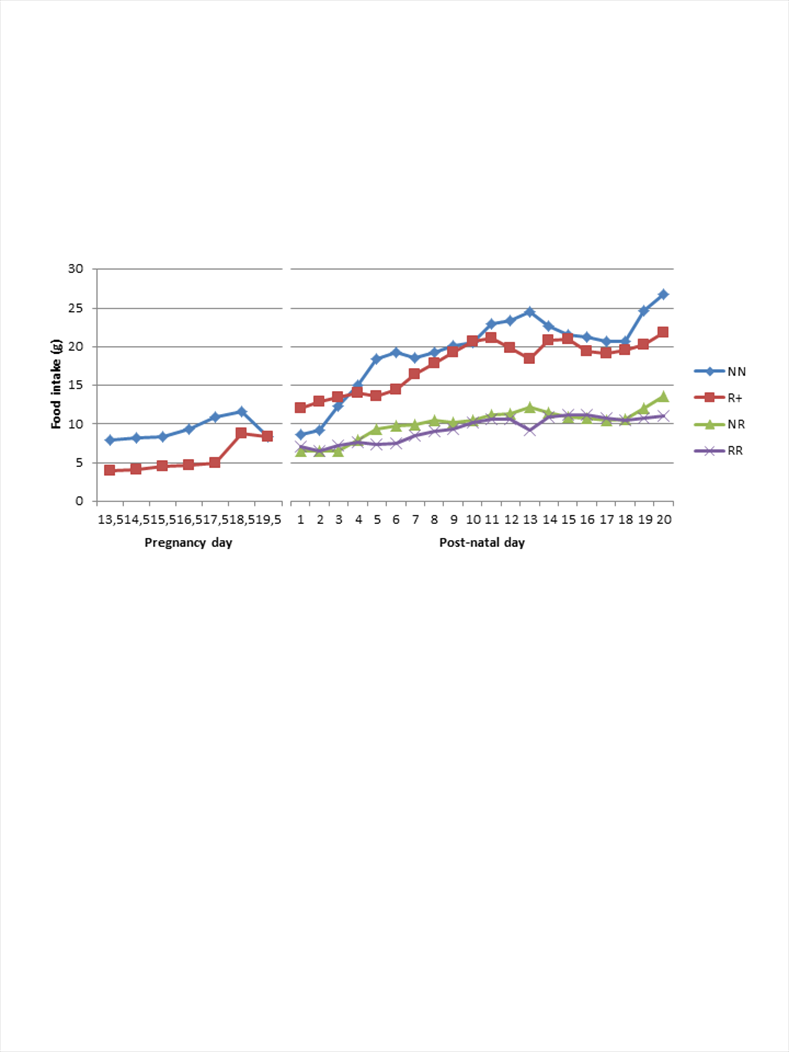

Supplement: S1 Fig — R+ and RR females received 50% of the food consumed compared to the average quantity received by the NN females. (TIF) [file pone.0177468.s001.tif]
